# The mental health and wellbeing outcomes and mechanisms of change in arts inclusive programs for children aged 6–12: a systematic review

**DOI:** 10.3389/frhs.2026.1827784

**Published:** 2026-06-26

**Authors:** Fotini Vasilopoulos, Eliza Oliver, An Nguyen, Isobel Ivison, Louise Birrell, Deanna Varley, Kirsty Rowlinson, Yihan Sun, Elloyse Saw, Olivia Karaolis, Robyn Ewing, Michael Anderson, Maree Teesson, Emma L. Barrett

**Affiliations:** 1 The Matilda Centre for Research in Mental Health and Substance Use, Faculty of Medicine, The University of Sydney, Camperdown, NSW, Australia; 2 The Create Centre, Faculty of Arts and Social Sciences, The University of Sydney, Camperdown, NSW, Australia; 3 School of Education, The University of Notre Dame, Sydney, NSW, Australia; 4 School of Public Health and Preventive Medicine, Monash University, Melbourne, VIC, Australia

**Keywords:** arts, childhood, mechanisms, mental health, wellbeing

## Abstract

**Background:**

Arts-based programs have re-emerged as a focus of population mental health policy, with the World Health Organisation Healing Arts initiative and social prescribing programs across the UK, Australia, USA, and Canada signaling growing interest in scalable, accessible approaches to mental health prevention. With this growing trend there is an opportunity for seeking complementary scalable approaches implemented during childhood to prevent mental-ill health.

**Objective:**

The aim of this study was to provide a broad overview of the nature of the relationships between participation in arts programs and mental health and wellbeing outcomes during middle childhood, with a specific emphasis on the mechanisms that may underlie this relationship. This review also aimed to identify program characteristics and the context in which this relationship exists.

**Methods:**

This systematic review was performed according to PRISMA guidelines with a focus on studies examining the relationships between arts participation and mental health and wellbeing outcomes among children in the general population during the middle childhood years (6 to 12 years).

**Results:**

A total of 34 studies were included, made up of primarily quantitative studies. The evidence base reviewed suggests therapeutic approaches in non-clinical settings led by specialists over shorter periods of implementation (typically 4–10 weeks) shows preliminary signs of promise. Recreational programs showed inconsistent effects across outcome domains: some studies reported benefits to wellbeing and stress, while others reported no effect on internalising or externalising symptoms, with no consistent pattern across program features. Psychological and biological constructs were the most commonly identified mechanisms of change. The certainty of evidence across all mental health outcomes was rated as low or very low.

**Conclusion:**

This first systematic review of arts-inclusive programs in middle childhood points to preliminary signs of promise, particularly for therapeutic approaches in non-clinical settings, although the certainty of evidence is low. Future research should focus on conducting high quality studies in school contexts with a focus on pedagogical design and facilitator expertise. This would address cost effectiveness for scaling at the general population level for prevention.

**Systematic Review Registration:**

https://www.crd.york.ac.uk/PROSPERO/view/CRD42024581364.

## 1 Introduction

Mental health disorders are a global public health priority, with over one third of mental health disorders occurring before the age of 14 (1). The World Health Organization (WHO) has identified children's mental health as a global priority and called for a shift toward community-based programs, recognising that “health” is not merely the absence of disease but a state of complete social and mental wellbeing (2). In this context, there is an urgent need to identify novel, effective and scalable solutions that prevent onset or reduce the severity of mental ill-health earlier in life. An accessible medium, the arts, is being reframed as a possible tool for improving population mental health. This has led to the rise of social prescribing in countries including the United Kingdom, United States of America, Australia and Canada (3–5). Social prescribing refers to health professionals formally prescribing non-clinical activities, including visual arts, music, and dance, to address social determinants of health such as loneliness and trauma (6). With this growing trend there is an opportunity for complementary scalable approaches implemented during childhood to prevent mental-ill health.

Middle childhood (6–12 years) is an often neglected but critical period of development during which emotional, cognitive, and social capacities undergo substantial consolidation (6, 7). There is emerging evidence that children's and young people's engagement in arts programs can positively impact their wellbeing [e.g., (8, 9)]. Engaging with art practices can be through actively creating or passively experiencing art as created by others (10). Previous reviews summarising the benefits of arts programs on mental health have focused on broader age groups of children and young people [e.g., 4–25 years: (9); 11–18 years: (11)]. However, evidence of the mental health benefits of engaging in the arts during the primary school years has not been systematically reviewed. In addition, it remains unclear which types of arts programs may benefit middle childhood mental health, and under what conditions benefits are most likely to occur.

### 1.1 Aims

The current review aims to systematically review the mental health and wellbeing outcomes from participation in arts-inclusive programs for children aged 6–12 years in the general population in non-clinical settings. A secondary aim is to examine the mechanisms of change through which arts-inclusive programs may influence mental health and wellbeing outcomes. A final aim is to exploratory review program characteristics and contextual factors that relate to mechanisms identified.

This review will consider arts programs that are inclusive of all children. Arts-inclusive programs encompass a range of structured arts activities, including visual arts, music, dance, drama, and creative writing, delivered to groups of children. For the purposes of this review, we distinguish two dimensions of arts-inclusive programs. Setting refers to the institutional context in which a program is delivered, ranging from clinical settings (where children are referred for treatment of a diagnosed condition) to non-clinical settings such as schools, community organisations, and out-of-school-hours programs. Approach refers to how the arts activity itself is designed and delivered: a therapeutic approach draws on therapeutic theory, typically delivered by a trained arts therapist; a recreational approach is delivered by teachers, artists, or community facilitators without such therapeutic framing. The present review focuses on programs spanning both therapeutic and recreational approaches in non-clinical settings. It includes all art forms to enable a mechanism synthesis capable of identifying processes that are common across arts forms as well as those specific to particular modalities, which is not possible within single-modality reviews. Finally, mechanisms of change refer to the psychological, biological, social, or behavioural processes through which participation in an arts-inclusive program is theorised or shown to influence mental health and wellbeing outcomes.

## 2 Methods

This systematic review was performed according to PRISMA guidelines and preregistered on PROSPERO (No. CRD42024581364). The primary search included four electronic databases: Medline, PubMed, ProQuest, PsycINFO and Scopus. In addition, we also searched reference lists and citations of eligible studies and previous reviews and meta-analyses of this literature to identify any additional studies. The search strategy was developed to identify key terms related to (i) art inclusive programs, (ii), mental health and wellbeing and, (iii) children in the general population (see Supplementary Table S1 for complete search terms and strategy). The search was restricted to peer-reviewed publications written in English and published in the last ten years (01/01/2014 and 09/09/2024). The searches were completed on September 10th 2024. This timeframe reflects the period following the WHO Mental Health Action Plan 2013–2020 (12), which positioned mental health prevention as a global priority and stimulated growth in non-clinical, scalable approaches to children's mental health. This time frame captures the contemporary evidence base relevant to current policy and practice contexts.

The eligibility criteria were developed with reference to the PICO framework [Population, Intervention, Comparison, Outcome; (12)]. if Studies were eligible for the review they (1) evaluated art program with an objective outcome measure of mental health or wellbeing, (2) the study population included 6–12-year-old children, (3) were peer-reviewed journal articles, and (4) were published in the English. Studies were included with or without a comparison group to capture a breadth of understanding from quantitative and qualitative studies. Programs delivered in either group or individual (1:1) formats were eligible for inclusion. Studies that applied one single session of arts exposure in less than two hours were excluded. This threshold was applied to distinguish structured arts programs from single-exposure or one-off activities, which are unlikely to engage the sustained psychological, social, or biological processes through which arts engagement is theorised to influence mental health. See Supplementary Table S2 for a complete list of inclusion and exclusion criteria.

**Figure 1:**
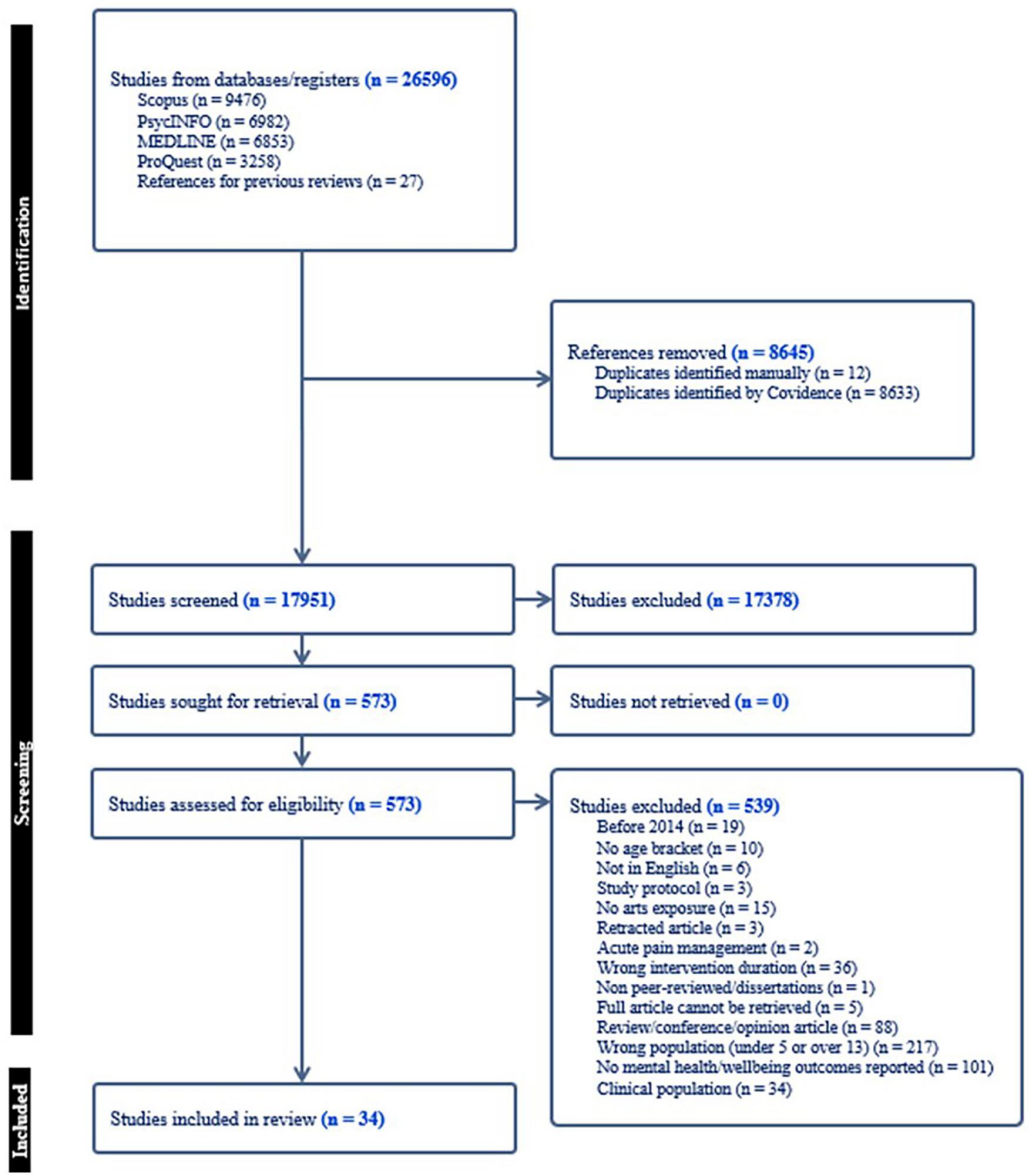
PRISMA flow diagram.

The search retrieved 17,951 unique articles, title and abstracts of these studies were then screened for eligibility with 573 articles progressing to full text screening. Full texts were then assessed, after which 34 articles were considered eligible for inclusion (Figure 1). Double reviewer screening was performed at both stages by a number of reviewers (FV, EO, AN, LB, DV, KR, ES, OK). Disagreements at both stages were resolved through discussion between the two reviewers until consensus was reached, with a third reviewer consulted when consensus could not be reached.

### 2.1 Data extraction

The quality of the included studies was assessed by two independent reviewers (II, YS) using the Mixed Methods Appraisal Tool 2018 [MMAT; (13, 14)], and any discrepancies were resolved with the involvement of a third reviewer (FV). Inter-rater reliability ranged from moderate to perfect across MMAT criteria (*κ* = 0.56 to 1.00, mean *κ* = 0.91) (Supplementary Table S3). Only one item on the MMAT criteria had an inter-rater reliability score of moderate (“*Is randomisation appropriately performed”)*. This was due to difficulty in applying the criterion in a consistent way based on the information provided in the relevant studies. An overall agreement score was 96.3%. The certainty of the evidence was assessed for each outcome using the Grading of Recommendations Assessment, Development and Evaluation (GRADE) approach (15). Assessment of quantitative studies included: risk of bias, inconsistency of results, indirectness of evidence, imprecision and publication bias. Assessment of qualitative studies included: methodological limitations, relevance, coherence and data adequacy (16).

Data extraction was completed by one reviewer (EO, AN, YS) and a second reviewer (II) independently audited the extracted data against the original studies to ensure reliability. Disagreements were settled through triangulation with a third reviewer (FV). A standardised data extraction form was used to record the following: (1) publication details, (2) participant demographics, (3) intervention characteristic (recreational or therapeutic), (4) program characteristics (frequency, number of sessions, duration, setting, trainer) (5) study methodology, (6) outcome measures, and (7) descriptive statistics of the outcome measures. A separate standardised protocol was developed to extract mechanisms, discussed in further detail below. Standardised effect sizes were calculated for studies with sufficient data reported using Campbell Collaboration online calculator (17). The SWiM guidelines have been used to guide our narrative synthesis of studies (18). Direction of effect was used to classify outcome impact as beneficial, harmful or none. In our narrative synthesis we prioritised well conducted studies (i.e., randomised control trials), certainty of evidence and relevance of the evidence addressing the review question (19).

Mechanisms were systematically extracted by one reviewer (FV) from all studies using a framework synthesis approach (20). The Multi-level Leisure Mechanisms Framework was used to organise mechanisms into psychological, biological, social, and behavioural domains (21). We made minor adaptations where developmentally relevant mechanisms emerged. For example, sleep quality was identified as a mechanism in one study and classified under biological mechanisms, as sleep regulation involves HPA axis and autonomic nervous system function (biological processes included in Fancourt's framework) and is particularly relevant to child development and mental health (22). We distinguished between tested mediators (constructs subjected to formal mediation analysis), qualitative mechanisms (processes described through participant or researcher accounts), and theoretical mechanisms (pathways proposed but not empirically tested). Constructs measured as outcomes but not formally tested as mediators were not classified as mechanisms, even when studies measured multiple related outcomes simultaneously. Mechanism type and contextual factors were extracted by one reviewer. A second reviewer (EO) independently extracted mechanisms from seven randomly selected studies (20% of total). Agreement on mechanism identification was high (82%), with discrepancies resolved through discussion. For each identified mechanism, we developed Context-Mechanism-Outcome (CMO) configurations following realist synthesis principles (23). We have chosen a realist synthesis approach because it allows us to understand what programs may work, how they work and in what circumstances (23).

## 3 Results

### 3.1 Characteristics of studies

A total of 34 studies were included in this systematic review (Figure 1; *n* = 27 quantitative, *n* = 3 qualitative; and *n* = 4 mixed methods). There were 23 studies that included control groups (*n* = 4 active control groups and *n* = 19 passive control groups), with a total of 4,207 participants across all studies. Of the studies that reported gender (60% of studies), 20 out of 34 included both sexes (mean percentage girls = 33%). Studies were conducted in 14 countries: United Kingdom (*n* = 8), Canada (*n* = 6), South Korea and USA (*n* = 4), Italy and Portugal (*n* = 2), Belgium, Brazil, Czech Republic, Greece, Hong Kong, India, Israel and Ukraine (*n* = 1).

### 3.2 Arts inclusive programs

**Figure 2:**
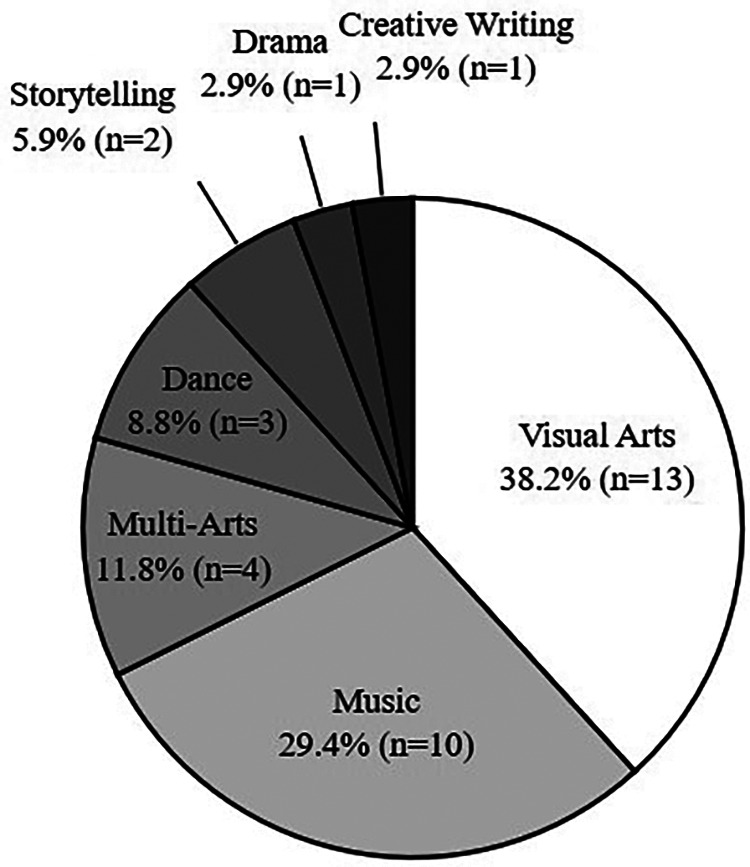
Distribution of arts activities Among included studies.

The majority of studies included therapeutic art (*n* = 23). Over one third of the programs implemented visual arts (*n* = 13; Figure 2). The duration of arts inclusive programs ranged from two weeks to 40 weeks, with the duration of approximately one third falling between 8 and 10 weeks (*n* = 11, 32%). Over half of the studies were conducted in a school setting (*n* = 21), with the remainder conducted in out-of-school hours programs (*n* = 3), community settings (*n* = 3), online via video conferencing (*n* = 2), home settings (*n* = 2), a clinic (*n* = 1), and a camp (*n* = 1). In one case, the setting was not reported (*n* = 1). Most studies measured outcomes immediately following the program (83%). Detailed characteristics of included studies are provided in the supplementary material Table S4. The studies used a range of outcome measures, which are summarised in Supplementary Table S5.

### 3.3 Impacts of arts practices on mental health and wellbeing outcomes in children

#### 3.3.1 Critical appraisal of studies

Results of the critical appraisal of studies examining the general population, using the MMAT 2018, are shown in Supplementary Figures S4, S5. The overall quality of quantitative studies and quantitative elements of mixed methods studies varied but was generally strong (see Supplementary Figure S6). However, a common limitation was the lack of outcome assessors blinded to the intervention condition (Supplementary Figure S7). Half of the studies included samples that were representative of the general population. Qualitative studies and qualitative elements of mixed methods studies (*n* = 11) were rated as strong on all aspects including, data sources, collection and analysis. Interpretation of findings were grounded in the original data and synthesised with quantitative elements for mixed methods studies.

#### 3.3.2 Mental health and wellbeing outcomes

**Figure 3:**
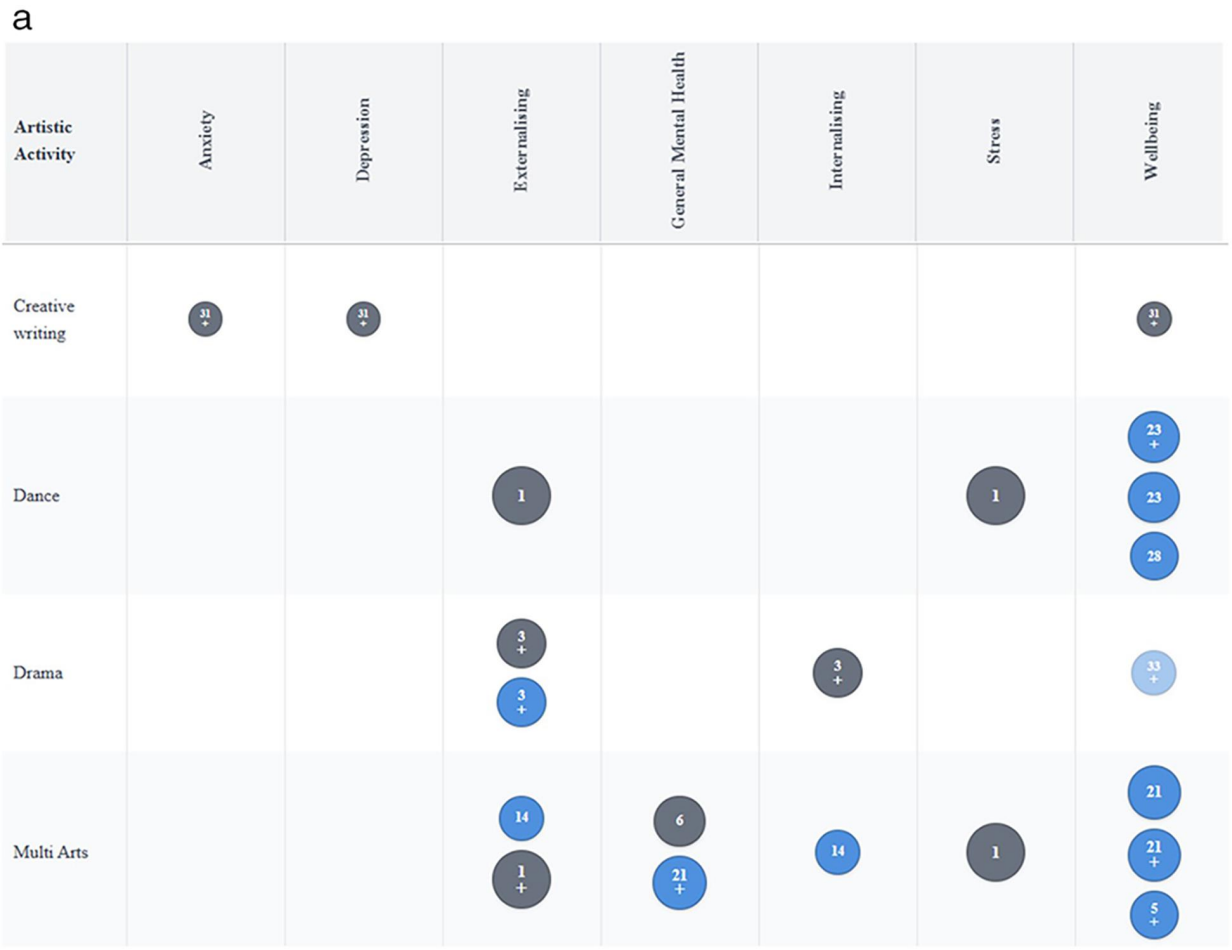
**(a,b)** Arts-based activities and mental health outcomes in children. Numbers refer to each study identified in supplementary material Table S3. Bubble colour: delivery approach; Blue = Therapeutic, Grey = Recreational. Opacity/Shade: type of study; Faded = Qualitative, Full = Quantitative. Symbol: whether the program showed beneficial effects; + = Beneficial, - = Harmful, Blank = No effect. Bubble size: sample size. 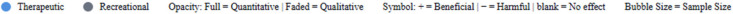.

Detailed information about the impact of arts inclusive programs on mental health and wellbeing outcomes according to activity is presented in Figures 3a,b and discussed below. We report outcomes based on Hierarchical Taxonomy of Psychopathology (24). Outcomes were measured with a variety of instruments. The Strengths and Difficulty Questionnaire was the most commonly used instrument to measure mental health and wellbeing (*n* = 6). Outcomes are presented from clinical mental health conditions (anxiety, depression), to behavioural dimensions of mental health (internalising and externalising symptoms), to states of wellbeing (stress, wellbeing), reflecting a progression from symptom-focused to wellbeing-focused outcomes. For the purposes of this review, wellbeing refers to positive psychological functioning (e.g., EQ-5D-Y, KIDSCREEN, Stirling Children's Wellbeing Scale), whereas general mental health refers to composite indicators of overall psychological functioning that incorporate both positive and problematic dimensions (e.g., SDQ Total Difficulties).

**Figure:**
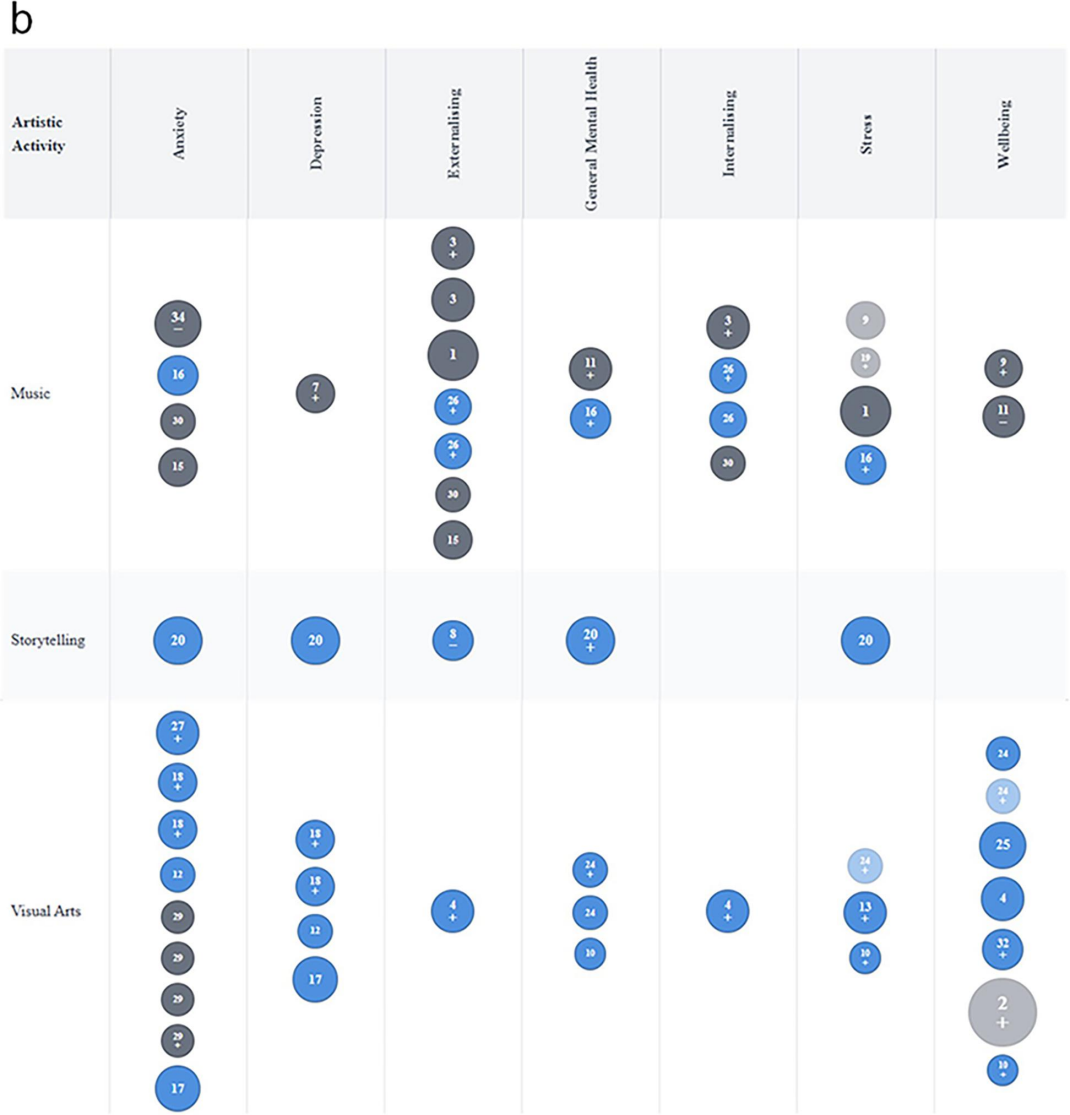


##### 3.3.2.1 Anxiety

Eleven studies assessed the impacts of arts inclusive programs on anxiety symptoms (Figure 3a). There was limited evidence that arts inclusive programs were associated with a reduction in anxiety symptoms (24–26). Based on the GRADE approach (Supplementary material Table S8), the certainty of the evidence whether arts inclusive programs improved anxiety symptoms was low. All studies used quantitative instruments to measure anxiety. Instruments used to measure anxiety varied, with the BASC-3 Anxiety subscale and Revised Children's Manifest Anxiety Scale used in two studies each (24, 26–28).

###### 3.3.2.1.1 Controlled Studies

In five well-conducted studies where the direction of effect could be determined (24, 25, 29–31), anxiety was lower in the intervention group compared to the control group after the arts inclusive programs in two studies [Figure 3b: (24, 25)]. Both studies implemented therapeutic visual arts by a researcher with structured instructions over a three-week period. In the remaining three studies (all of which were randomised control trials), there was no benefit to anxiety outcomes between the intervention group and control group (29–31). Of these three studies, one implemented music with a therapeutic approach [Figure 3b: (30)], and two in a recreational context (29, 31). The recreational activities were school based over a long period of time (24–40 weeks) and taught by a music school teacher. Both took a different approach with one taking a teacher-led approach [Figure 3b: (29)] and the other a student-led approach [Figure 3a: (31)].

###### 3.3.2.1.2 Uncontrolled Studies

Anxiety outcomes in the remaining six studies were mixed. Three of these studies, two of which applied visual arts in a therapeutic [Figure 3b: (27, 28)] and one in a recreational approach, [Figure 3b: (32)] identified no associations between arts inclusive programs and anxiety. The visual art activities involved facilitator-led free drawing sessions implemented over 10–12 weeks. The final two reviewed studies implemented storytelling through verbal [Figure 3b: (33)] or written means [Figure 3b: creative writing (26)]. The study that implemented creative writing in a recreational context reported benefits in child anxiety (27), while the study that implemented therapeutic verbal storytelling reported no benefit on anxiety. In the last study, children enrolled in an intensive music program at school demonstrated increased in anxiety among girls [Figure 3b: (34)].

##### 3.3.2.2 Depression

Six studies assessed the impacts of arts inclusive programs on depressive symptoms. There was limited evidence that arts inclusive programs were associated with child depression outcomes (24, 26, 35). Based on the GRADE approach (Supplementary material Table S8), the certainty of the evidence whether arts inclusive programs improve depressive symptoms was low. All instruments used to measure depression were used once across studies. Notably, one of these instruments was a drawing task completed by children (28).

###### 3.3.2.2.1 Controlled Studies

In two well-conducted studies where the direction of effect could be determined, depression was lower in the intervention group than in the control group after the arts inclusive programs [Figure 3b: (24, 35)]. One study implemented therapeutic visual arts with structured instructions over a three-week period (25) with the other implementing a structured program of recreational music over a 24-week period for one hour per week by an artist (36).

###### 3.3.2.2.2 Uncontrolled Studies

In the remaining four studies, only one showed benefit in depressive symptoms [Figure 3b: (26)]. This is the only study that was implemented in a recreational means by a teacher in that profession (creative writing four times per week). The remaining three studies implemented therapeutic art practices facilitated by adults with varying degrees of qualifications (researcher, teacher or healthcare professional) over a similar number of sessions (8–12 weeks) and did not identify any meaningful benefit on depressive symptoms [Figure 3b:Visual arts (27, 28); Story telling (33)].

##### 3.3.2.3 Internalising symptoms

Five studies assessed the impacts of arts inclusive programs on internalising symptoms. There was mixed evidence that arts inclusive programs were associated with reductions in internalising symptom outcomes. Based on the GRADE approach (Supplementary material Table S8), the certainty of the evidence whether arts inclusive programs improve internalising symptoms was very low. All instruments used to measure internalising symptoms were used once across studies as rated by an adult.

###### 3.3.2.3.1 Controlled Studies

In four well-conducted studies (31, 36–38), where the direction of effect could be determined, internalising symptoms were lower in the intervention group than in the control group after the arts inclusive programs in two studies (36, 37). One study implemented two separate recreational artistic activities (drama and music) over a 30-week period delivered one hour per week by a school teacher in the relevant field (37). The sessions were based on active participatory pedagogies. The other program implemented therapeutic visual arts by an art therapist for eight weekly one-hour sessions with highly structured activities [Figure 3b: (37)]. The two remaining studies included randomised control trials where the intervention group and control group showed no benefit on internalising symptoms following the program (31, 38). One study delivered multi arts using a therapeutic approach [Figure 3a: (38)] or music in a recreational school context [Figure 3b: (31)]. Both studies implemented highly structured activities. The recreational music program in the latter study was school based over a long period of time (40 weeks, one hour per week) and taught by a music school teacher (32).

###### 3.3.2.3.2 Uncontrolled Studies

Impacts in the remaining study were mixed [Figure 3b: (39)]. Ten weekly sessions of structured music applying a therapeutic approach were delivered by a music therapist. Parents had rated that the sessions benefited internalising symptoms in contrast to teachers.

##### 3.3.2.4 Externalising symptoms

Eight studies assessed the impacts of arts inclusive programs on externalising symptoms. There was limited evidence that arts inclusive programs were associated with externalising symptom outcomes. The certainty of evidence that arts inclusive programs improve externalising symptoms was low (Supplementary material Table S8). Instruments used to measure externalising symptoms varied, however, the SDQ was used in two studies (38, 40).

###### 3.3.2.4.1 Controlled Studies

In five well-conducted studies where the direction of effect could be determined (29, 31, 36–38), externalising symptoms were lower in the intervention group than in the control group after the arts inclusive programs in the two same studies that also measured internalising symptoms [Figure 3b: (36, 37)]. In the three remaining randomised control trials, there were no beneficial changes in externalising symptoms between the intervention and control groups (29, 31, 38).

###### 3.3.2.4.2 Uncontrolled Studies

In the remaining three studies, one reported benefit in externalising symptoms following eight weeks of structured music therapy sessions facilitated by a music therapist [Figure 3b: (39)]. The remaining studies identified no benefit to externalising symptoms. The direction of effect for one study was considered harmful and implemented therapeutic storytelling facilitated once by a guest reader and then by parents three to five times per week for six weeks [Figure 3b: (41)]. There was no benefit in the other study, a single cross-sectional measurement of externalising symptoms across groups with a history of participating in dance, music or no prior participation in either activity [Figures 3a,b: (40)].

##### 3.3.2.5 Stress

Six studies assessed the impacts of arts inclusive programs on stress. There was mixed evidence that arts inclusive programs were associated with stress outcomes (Supplementary material Tables S8, S9: moderate certainty evidence). Instruments used to measure stress varied widely across studies, with approaches ranging from child-reported questionnaires to qualitative methods such as focus groups (focus groups (42, 43):; questionnaires (33, 40, 44, 45):.

###### 3.3.2.5.1 Controlled Studies

In two well-conducted studies where the direction of effect could be determined, stress was lower in the intervention group than in the control group after the arts inclusive programs [Figure 3b: Visual arts (46); Music (30)]. Both studies implemented a therapeutic approach by a healthcare professional with highly structured adult led activities across 8–10 sessions lasting 45 min each session.

###### 3.3.2.5.2 Uncontrolled Studies

In the remaining four studies, two reported benefits to stress [Figure 3b: Visual arts (44); Music (43)]. Both studies implemented adult-led, structured activities. The visual arts practice took a therapeutic approach facilitated by an art therapist in two-hour sessions taking place in an art centre for 10 weekly sessions (45). The music practice involved children listening to music in a recreational context, followed by collection of qualitative data via focus groups (44). The remaining studies identified no benefit in stress implemented therapeutic storytelling by teachers two times per week in 30 min sessions over eight weeks [Figure 3b: (33)] or in a single cross-sectional measurement of stress across groups with a history of participating in dance, music or multi arts participation [Figure 3a: (40)].

##### 3.3.2.6 Wellbeing

Fourteen studies assessed the impacts of arts inclusive programs on wellbeing. There was mixed evidence that arts inclusive programs were associated with wellbeing outcomes. Based on the GRADE approach (Supplementary material Tables S8, S9), the certainty of evidence for arts inclusive programs benefiting wellbeing was low. Instruments used to measure wellbeing varied across studies, however, one child self-report instrument was the most common measurement tool, used in three studies [EQ-5D-Y (QOL): (45–47)].

###### 3.3.2.6.1 Controlled Studies

In six well-conducted studies where the direction of effect could be determined, wellbeing was better in the intervention group than in the control group after the arts inclusive programs in two studies [Figure 3a: Multi arts (47); Dance (49)]. Both studies implemented a therapeutic approach in two-hour sessions once per week for 4–8 weeks in schools using a manual or led by a facilitator. A specialist facilitator (healthcare professional or art therapist) led the sessions. Of the four remaining randomised control trials, three identified no benefit in wellbeing between the intervention and control groups, after implementing structured, therapist-led visual art or dance activities in school settings for one hour per week for 8–12 weeks [Figure 3b:Visual arts (37, 45); Figure 3a: Dance (50)]. Interestingly, the remaining randomised control trial implemented a school-based recreational choir program and identified reductions in wellbeing [Figure 3b: (51)].

###### 3.3.2.6.2 Uncontrolled Studies

In the remaining seven studies, most reported benefits to wellbeing [Visual arts: (44, 52); Drama: (53); Music: (42); Creative writing: (26); Multi arts: (54)]. Four of these studies implemented therapeutic approaches facilitated by researchers, mainly in school settings, that involved structured sessions lasting one to two hours per week for durations ranging between 6 and 40 weeks (44, 52–54). The remaining three studies implemented structured recreational activities [Music: (42); Creative writing: (26); Visual arts: (55)]. One study implemented artist-facilitated therapeutic visual arts activities for one full day every week for eight weeks however no benefit in wellbeing were observed [Figure 3b: (48)].

##### 3.3.2.7 General mental health

Seven studies assessed the impacts of arts inclusive programs on overall mental health. There was mixed evidence that arts inclusive programs were associated with mental health outcomes. Based on the GRADE approach (Supplementary material Table S8), the certainty of the evidence in relation to whether arts inclusive programs benefit mental health was very low. The SDQ was the most frequently used tool to measure this outcome, having been used in approximately half of the reviewed studies (45, 47, 51, 56). Most studies used adult reports to measure overall mental health.

###### 3.3.2.7.1 Controlled Studies

In five well-conducted studies, where the direction of effect could be determined, four suggested that overall mental health was better in the intervention group compared to the control group following participation in arts inclusive programs [Figure 3b: Music (31, 52); Visual arts: (46); Figure 3a: Multi-arts: (47)]. Notably, all four of these studies involved delivery of structured sessions and three of the four studies implemented a therapeutic approach led by a trained specialist (e.g., art therapist or healthcare professional (31, 46, 48);. In contrast, the fourth study implemented a recreational, school-based program facilitated by teachers [Figure 3a: Multi-arts (56)].

###### 3.3.2.7.2 Uncontrolled Studies

Finally, while the remaining study, the direction of effect was beneficial in overall mental health after implementing a therapeutic, art therapist-led program [Figure 3b: Visual arts (44)].

#### 3.3.3 Mechanisms of change

Twenty unique mechanisms were identified across 11 studies (31%), distributed across psychological (*n* = 6, 55%), biological (*n* = 7, 63%), and social (*n* = 2, 13%) domains. All mechanisms were identified through qualitative themes emerging from participant/teacher reports or theoretically proposed pathways developed with reference to prior literature. No studies statistically tested mediation (Supplementary Table S10). Psychological (55%) and biological (63%) mechanisms were identified in the majority of qualitative studies which captured children's subjective experiences of art participation. Self-efficacy emerged as the most commonly reported psychological mechanism, identified through qualitative analysis across three different art practices [Visual arts: (49); Drama: (54); Music: (52)]. Children reported on their self-efficacy for example, “Art is about actually trying … if you’re bad, keep trying. Don't give up” (49) and “I would like to do more because I have never had this much confidence before and expressed my feelings to other people” (54). Other psychological mechanisms identified included: stress reduction, increased positive affect, emotional expression, self-esteem, coping skills, and meaning making. Overall, our results suggest there may be common psychological mechanisms across diverse artistic activities. Biological mechanisms identified included: sleep quality, reduced cortisol, relaxation, embodiment, and physical activity. Sleep quality was the most commonly reported behavioural mechanism, identified through quantitative analysis across three different art practices [Multi-arts: (48); Dance: (50); Visual arts: (46)]. Social mechanisms included social belonging and collaboration. No behavioural mechanisms were identified. Notably, social and behavioural mechanisms were underrepresented despite most interventions being group-based, possibly indicating either a gap in the literature wherein these mechanisms have not been sufficiently considered, or these being less common or less influential mechanisms underlying relationships between arts inclusive programs and mental health.

##### 3.3.3.1 Context-mechanism-outcome

The CMO analysis revealed distinct mechanism profiles associated with different intervention contexts (Supplementary Table S11). Recreational interventions (*n* = 5) primarily activated psychological mechanisms (increased positive affect, self-efficacy, coping) and social mechanisms (belonging, collaboration), while various psychological and biological mechanisms were suggested to underlie therapeutic intervention effects (*n* = 6). A discernible mechanism profile also emerged for music interventions (*n* = 4), with psychological and social mechanisms linking music to various mental health and wellbeing outcomes. This suggests that music's group-based nature may activate social bonding alongside affective responses. Wellbeing was the most common outcome identified in the CMO analysis, with mechanisms from the psychological, social and biological domains linked to wellbeing benefits. This suggests that there may be multiple pathways through which arts practices and wellbeing may be related.

## 4 Discussion

This systematic review examined the efficacy of arts inclusive programs across multiple mental health outcomes between the ages of 6–12 years. The evidence base included studies evaluating varied arts inclusive programs across domains including anxiety, depression, internalising and externalising symptoms, wellbeing and overall mental health. The findings had low to very low certainty of evidence, with mixed associations reported. Notably, stress reduction demonstrated the most consistent positive outcomes, with therapeutic visual arts and music interventions both producing significant reductions in stress in randomised controlled trials [Visual arts: (47); Music: (31)]. In contrast, anxiety, depression, internalising and externalising symptoms showed limited evidence. We also examined potential mechanisms of change and how context shapes these. Wellbeing was the most common outcome measured with all mechanisms related to wellbeing benefits.

### 4.1 Outcomes of arts inclusive practices

The first aim of our study was to systematically review the mental health and wellbeing outcomes associated with participation in arts-inclusive programs for children aged 6–12 years in non-therapeutic settings. We identified considerable heterogeneity in artistic activity, program characteristics and the expertise of those facilitating the program across the reviewed studies, and associations between arts practices and mental health outcomes. For example, benefits on wellbeing were observed for programs that involved implementation of therapeutic approaches delivered by qualified specialists (art therapists, music therapists, healthcare professionals) over periods ranging from four to ten weeks. Further, arts inclusive programs were linked to benefits in general mental health outcomes in studies applying therapeutic approaches led by trained specialists (e.g., art therapists) in a structured format (e.g., manualised format). In contrast, recreational programs facilitated by teachers in school settings typically showed no benefit with student mental health or wellbeing outcomes.

However, these findings should be regarded as preliminary. Considerable heterogeneity in program design, delivery context, and measurement approaches, combined with low-to-very-low certainty of evidence, precludes our ability to draw definitive conclusions regarding the impact of arts inclusive programs for improving child mental health and wellbeing outcomes. Establishing whether characteristics such as artistic modality, delivery context, or facilitator expertise genuinely moderate outcomes will require a larger and more homogeneous body of evidence than is currently available. As studies differed across several characteristics at once, apparent advantages such as those of specialist-led delivery cannot be separated from co-occurring differences in setting, structure, or duration. Any patterns we describe across studies should therefore be read as tentative observations rather than moderating effects, as too few studies fell within any given combination of outcome, modality, and context to support formal subgroup comparison.

The included studies also share methodological limitations that impact the likely direction of bias. Blinding of participants, facilitators, and assessors was frequently absent, raising the possibility that bias inflated observed benefits. Several studies also lacked a control group, making it difficult to distinguish program effects with most assessed outcomes only in the short term, when novelty and engagement effects may be strongest.

A further consideration concerns how the included evidence aligns with the prevention orientation of this review. Although some programs drew on therapeutic approaches, such as art or music therapy, the basis on which children were enrolled, rather than the approach itself, determined where a program sat within a prevention framework. The majority of included programs (27 of 34) are best characterised as universal prevention, being open to all children within a school or community setting irrespective of individual risk (57). Six studies reflect selective prevention, targeting groups at elevated risk on the basis of shared circumstance rather than diagnosed need. For example, children living in areas of high socioeconomic deprivation or siblings of children with disabilities (28, 36, 39, 47, 49, 53).

Only one study met the threshold for indicated prevention, which targets children already showing early symptoms (50), and none delivered treatment to clinically diagnosed populations, consistent with our setting-based eligibility criteria. Viewed this way, even programs employing therapeutic arts approaches operated as universal or selective prevention when delivered to general-population children in non-clinical settings, rather than as indicated treatment. The distinction that matters for prevention is the population reached and the basis for participation, not the therapeutic lineage of the activity. Our review therefore suggests the potential of arts-inclusive programs as universal and selective preventive strategies, while indicating that their value as indicated prevention remains largely untested.

Where described, a majority of studies implemented structured, adult-led sessions to facilitate sessions (*n* = 29). In contrast, child-led pedagogical frameworks in programs were rare. Two studies which applied a child-led framework showed preliminary signs of promise for reducing internalising symptoms (32, 37). These frameworks would arguably be less procedurally burdensome on generalist teachers than implementing a manualised approach because it gives teachers pedagogical agency reducing the risk of intervention decay in primary school settings. However, both of the studies implemented their approach over a long-period of time (30-weeks or more). It is possible benefits take time to be realised and it is unknown whether significant effects would be observed if delivery was implemented over a shorter period of time. Without a control group, it is challenging to determine whether observed changes can be attributed directly to the program.

In summary, in relation to aim one, some arts inclusive programs showed some potential for improving children's mental health, in particular stress and wellbeing. However, considerable heterogeneity and low certainty of evidence, mean that definitive conclusions or casual links could not be established. More rigorous research, including longer-term follow up and robust design, are needed before recommending arts inclusive programs as an effective prevention strategy for mental health and wellbeing during childhood.

### 4.2 Mechanisms of change

The second aim of this review was to examine mechanisms of change. Psychological and biological mechanisms were identified as the most prevalent. Psychological factors, particularly self-efficacy, were associated with both practicing artistic endeavours and mental health (49, 52, 54). In mental health research**,** self-efficacy is also sometimes referred to as enactive attainment (58). Enactive attainment involves someone building psychological capital in a low stakes environment to be able to face challenges later (58). When one is involved in the artistic process, a participant has physical proof of their agency and they can take creative and social risks in a safe space inclusive of diverse skill levels (59). This may thereby facilitate increased self-efficacy/enactive attainment, which may in turn have mental health benefits. Our findings are also consistent with three reviews that also identified psychological mechanisms such as self-efficacy and emotional expression when adolescents participate in arts inclusive programs (9, 11, 60). This consistency between our findings and these previous reviews suggests the possibility that psychological pathways may be developmentally universal mechanisms underlying the positive associations between arts participation and mental health and wellbeing.

However, a key distinction between reviews focusing on adolescence and our study is the use of biological instruments used to identify potential biological mechanisms (61, 62). Although biomarker dysregulation is more pronounced during adolescence (63), previous reviews appear to indicate that studies focused on adolescence may less frequently feature biological instruments compared to studies focusing on middle childhood as identified in this review. While it is possible that this may reflect methodological differences between reviews, this could also be due to ease of administering such instruments in a younger cohort compared to self-report instruments.

Our review of possible mechanisms of change also highlighted several gaps in the literature. Notably, behavioural mechanisms were unexplored, despite their theoretical importance during child development. In addition, studies reviewed here did not statistically test mediation, limiting causal inference. While our findings are consistent with an adult-facilitated structure being appropriate for middle childhood, reviews in adolescence emphasise the importance of social networks [e.g., (9)]. Future research could focus on social mechanisms when implementing art-inclusive programs for primary school aged children.

### 4.3 Contextual factors

Overall, we did identify some patterns linking program characteristics to mental health or wellbeing outcomes through various mechanistic pathways. First, recreational interventions primarily involved psychological and social mechanisms. This aligns with evidence suggesting children experiencing social-emotional difficulties may respond well to recreational community programs [e.g., (64)]. Second, music interventions benefited mental health and wellbeing outcomes via social and psychological/affective pathways. Group music-making inherently requires coordination, turn-taking, and collective creation, organically fostering social connection. Given this, recreational group music programs may offer particularly useful interventions through social mechanism pathways for children experiencing social isolation or difficulties with peer relationships.

### 4.4 Implications for future research and practice

This review points to several priorities for future research and practice. The mechanisms through which arts-inclusive programs influence mental health and wellbeing remain largely untested. Of the studies reviewed, mechanisms were proposed theoretically or identified as qualitative themes, and none employed formal mediation analysis. The mechanistic focus should be more on social and behavioural mechanisms, which were underrepresented or absent, despite arts participation being inherently social and behavioural. Gold standard RCTs are required to improve the quality of evidence to draw definitive conclusions regarding the impact of artistic programs arts inclusive programs for improving child mental health and wellbeing outcomes.

The predominance of structured programs also leaves open whether more child-led, exploratory approaches confer comparable or distinct benefits, warranting research that varies the degree of child-led participation. Longer follow-up is needed to determine whether benefits are sustained or emerge over time, with direct implications for prevention and early-intervention framing. Also, none of the included studies reported cost data or economic evaluation, and establishing the cost-effectiveness of these programs, particularly when delivered in community and educational settings, would strengthen the case for investment. Finally, as this review was restricted to English-language publications concentrated in high-income settings, future syntheses and primary studies extending to non-English-language contexts and lower- and middle-income countries would improve generalisability and support the equitable development of arts-inclusive programs across diverse settings.

### 4.5 Strengths and limitations

A strength of this study is that it is the first systematic review to focus on the primary school years when understanding the relationship between undertaking artistic endeavours and mental health. A further strength is the synthesis of mechanisms of change, which moves beyond cataloguing outcomes to consider how arts-inclusive programs may support mental health and wellbeing. Identifying these mechanisms provides actionable insight for stakeholders at multiple levels, from practitioners designing programs to policymakers determining where to direct resources, by clarifying not only whether arts programs work but the pathways through which their benefits may arise, although we note the quality of evidence means there is high uncertainty in the mechanisms identified. In the current study, we focused on the general population to support scalability of prevention.

Although the risk of bias was generally low, a common limitation was the lack of blinding of outcome assessment and intervention delivery, which can be difficult to implement in education settings. All studies meeting our eligibility criteria used group delivery formats, despite no restriction in our protocol on delivery format. The synthesis therefore cannot inform conclusions about the effects of individually delivered arts-inclusive programs. The review was restricted to English-language publications, which may have excluded relevant evidence from non-English-speaking contexts and limits the generalisability of findings across diverse settings. Our eligibility criteria also encompassed a broad range of arts-inclusive programs, which, while capturing the breadth of the field, limits direct comparability across forms. The ten-year search window was selected to align with contemporary policy and methodological developments but excludes foundational earlier studies, particularly from the arts therapies literature of the 1990's and 2000's. Another limitation includes not accounting for differences in the relative size of studies when synthesising effect sizes.

## 5 Conclusion

Given the growing concerns for the mental wellbeing of young people, seeking complementary scalable approaches to prevent mental-ill health during middle childhood is warranted. This is the first systematic review to investigate the relationship between participation in arts inclusive practices and mental health during middle childhood. Based on our findings, therapeutic approaches in non-clinical settings led by specialists over shorter periods show preliminary signs of promise. However, the conclusions are limited by low-to-very-low certainty evidence and the absence of formal mechanism testing. An opportunity for high quality studies that take a recreational approach conducted in school settings is warranted as evidence of such approaches remains limited. These approaches could also address issues related to cost effectiveness for prevention. Our review suggests that art-related programs may provide a promising opportunity for this although further high-quality evidence is needed.

## Data Availability

The original contributions presented in the study are included in the article/Supplementary Material, further inquiries can be directed to the corresponding author.
