# Supplementary material for: The mental health and wellbeing outcomes and mechanisms of change in arts inclusive programs for children aged 6–12: a systematic review
Source: Front Health Serv. 2026 Jun 26;6:1827784. doi: 10.3389/frhs.2026.1827784 (PMC13350468; doi:10.3389/frhs.2026.1827784)
Supplement: Supplementary file 1 [file Table1.docx]

**Supplementary Table S1**. Complete Search Strategy

| **MEDLINE** | ("mental health" or "behavioural problem*" or "behavioral problem*" or "emotional problem*" or "social problem*" or depress* or mood or internalizing or internalizing or sad* or low or unhapp* or Anxi* or internalizing or internalizing or worr* or Conduct or aggress* or "oppositional defiant disorder*" or "oppositional defiant" or oppositional or externalizing or externalizing or misbehaviour or misbehavior or fight* or cheating or violen* or defian* or ang* or argu* or attention or "attention deficit" or hyper* or concentrat* or autis* or "autism spectrum disorder" or asperg* or "emotion dysregulation" or "emotion regulation" or "emotional regulation" or "affect regulation" or "self regulation" or tantrum* or outburst* or meltdown or "emotion coaching" or "social skill*" or "friendship problem*" or bully* or bulli* or "interpersonal problem*" or "social problem*" or "peer problem*" or "peer relation*" or "social relation*" or connection or "peer skills" or "prosocial" or Attachment or "parent-child relationship*" or "parent-child connection*" or "attachment style*" or "reactive attachment" or "secure attachment" or "insecure attachment" or "avoidant attachment" or "attachment rupture" or "dismissive attachment" or "disorganised attachment" or "disorganized attachment" or Trauma or "post-traumatic stress disorder" or "posttraumatic stress disorder" or "trauma-informed" or trauma or stress or "adverse child experience*" or divorce or war or assault* or abuse* or neglect or "household dysfunction" or "physical abuse" or "emotional abuse" or "sexual abuse" or "physical neglect" or "emotional neglect" or "mental illness" or "incarcerated relative" or "mother treated violently" or "domestic violence" or "substance abuse" or "sensory-regulation" or "sensory processing difficult*" or "sensory processing disorder*" or "physical wellbeing" or "physical well being" or feeding disorder* or eating disorder* or motor disorder* or neurotic disorder* or personality disorder* or neurodevelopmental disorder* or neurocognitive disorder* or rape* or tortur*), (Art* or creativ* or theatre or theater or drama* or acting or music or sing* or craft* or drawing* or paint* or danc* or "musical instrument" or reading or litera* or "creative writing" or sculpture or sewing or "film making" or "movie making" or design or choir or film or band or orchestra or mythology* or poetry* or poem* or creative art* or colouring* or coloring* or cultural* or multimedia* or illustrate* or sketching* or photography*), (child* or middle childhood* or "age 2 - 5" or "age 6-12" or school age* or school-age* or elementary school* or primary school*) |
| --- | --- |
| **Scopus** | ( TITLE-ABS-KEY ("mental health" OR "mental problem*" OR "mental disorder*" OR "emotional health" OR "behavio?r* problem*" OR "emotional problem*" OR depress* OR mood OR unhapp* OR Anxi* OR internali?ing OR worry* OR "Conduct disorder*" OR aggress* OR "oppositional defiant" OR externali?ing OR misbehaviour OR violen* OR defian* OR "emotion* dysregulation" OR "emotion* regulation" OR "emotion* regulation" OR "affect* regulation" OR self?regulation OR tantrum* OR outburst* OR meltdown OR stress* OR coping OR distress* OR "disinhibited social engagement disorder" OR "reactive attachment disorder" OR "psychosocial outcome*" OR "psychosocial functioning" OR psychopatholog* OR suicid* OR self?injur OR self?harm* OR wellness OR "positive affect*" OR "negative affect*" OR "panic attack*" OR "affective symptom*" OR catastrophi?ation* OR "psychological issue*" OR "self?destructive behavio" ) AND TITLE-ABS-KEY ( arts OR creativ* OR theatre* OR theater* OR drama* OR acting* OR music* OR singing OR craft* OR handicraft* OR craft-making OR craftmaking OR drawing* OR painting* OR dance* OR dancing OR "musical instrument" OR reading OR "creative#writing" OR sculpture* OR sculpturing OR sewing OR "film#making" OR "movie#making" OR "participatory video" OR designing OR choir OR video#recording OR orchestra OR poetry OR poem* OR creative AND art* OR colo#ring* OR multimedia* OR illustrating* OR sketching* OR photography OR "photographic art*" OR story#telling ) AND TITLE-ABS-KEY ( child OR children OR "middle childhood*" OR "age 6-12" OR "school#age*" OR "elementary#school*" OR "primary#school*" OR grade#school* ) AND TITLE-ABS-KEY ( program* OR intervention* OR activit* OR prevent* OR training OR initiative* OR approach* OR therap* ) ) AND PUBYEAR > 2003 AND ( LIMIT-TO ( LANGUAGE , "English" ) ) |
| **Psychinfo** | ("mental health" or mental problem* or mental disorder* or emotional health or "behavio#r* problem*" or "emotional problem*" or depress* or mood or unhapp* or Anxi* or internali#ing or worry* or Conduct disorder* or aggress* or "oppositional defiant" or externali#ing or misbehaviour or violen* or defian* or "emotion* dysregulation" or "emotion* regulation" or "emotion* regulation" or "affect* regulation" or "self#regulation" or tantrum* or outburst* or meltdown or stress* or coping or distress* or "disinhibited social engagement disorder" or "reactive attachment disorder" or psychosocial outcome* or "psychosocial functioning" or psychopatholog* or suicid* or self#injur or self#harm* or wellness or positive affect* or negative affect* or panic attack* or affective symptom* or catastrophi#ation* or psychological issue* or self#destructive behavio#r* or emotion* disturbance* or self#mutilation or psychiatric disorder*), (Well-being or welbeing or wellbeing or well being), (Arts or creativ* or theatre* or theater* or drama* or acting or music* or singing or craft* or handicraft* or craft-making or craftmaking or drawing* or painting or dance* or dancing or "musical instrument" or reading or "creative writing" or sculpture* or sculpuring or sewing or "film#making" or "movie#making" or "participatory video" or designing or choir or video#recording or orchestra or poetry or poem* or creative art* or colo#ring* or multimedia* or illustrating* or sketching* or photography or "photographic art" or story#telling), (child or children or middle childhood* or "age* 6-12" or school#age* or elementary school* or primary school* or grade school*), (program* or intervention* or activit* or prevent* or training or initiative* or approach* or therap*) |
| **ProQuest** | summary("mental health" OR "mental problem*" OR "mental disorder*" OR "emotional health" OR "behavio?r* problem*" OR "emotional problem*" OR depress* OR mood OR unhapp* OR anxi* OR internali?ing OR worry* OR "Conduct disorder*" OR aggress* OR "oppositional defiant" OR externali?ing OR misbehavio?r OR violen* OR defian* OR "emotion* dysregulation" OR "emotion* regulation" OR "emotion* regulation" OR "affect* regulation" OR self?regulation OR tantrum* OR outburst* OR meltdown OR stress* OR coping OR distress* OR "disinhibited social engagement disorder" OR "reactive attachment disorder" OR "psychosocial outcome*" OR "psychosocial functioning" OR psychopatholog* OR suicid* OR self?injur OR self?harm* OR wellness OR "positive affect*" OR "negative affect*" OR "panic attack*" OR "affective symptom*" OR catastrophi?ation* OR "psychological issue*" OR "self?destructive behavio" OR "emotion* disturbance*" OR self?mutilation OR "psychiatric disorder*" OR delinquenc* OR hyperactiv* OR antisocial OR "biosocial interaction*" OR "child behavio*r disorder*" OR "peer problem*" OR "conduct problem*") AND summary(arts OR creativ* OR theatre* OR theater* OR drama* OR acting* OR music* OR singing OR craft* OR handicraft* OR craft-making OR craftmaking OR drawing* OR painting* OR dance* OR dancing OR "musical instrument" OR reading OR creative?writing OR sculpture* OR sculpturing OR sewing OR film?making OR movie?making OR "participatory video" OR designing OR choir OR video?recording OR orchestra OR poetry OR poem* OR creative AND art* OR colo?ring* OR multimedia* OR illustrating* OR sketching* OR photography OR "photographic art*" OR story?telling) AND summary(child OR children OR "middle childhood*" OR "age 6-12" OR school?age* OR elementary?school* OR primary?school* OR grade?school*) AND summary(program* OR intervention* OR activit* OR prevent* OR training OR initiative* OR approach* OR therap*) AND (la.exact("ENG") AND pd(20040101-20241231) AND PEER(yes)) AND PEER(yes) |

**Supplementary Table S2** *Inclusion and exclusion criteria*

|  | **Include** | **Exclude** |
| --- | --- | --- |
| **Population** | Primary-school aged children (5-13 years) | Under 5 years, over 13 years  Populations where these age groups are not reported out separately i.e., “young people under 25”. |
| **Intervention** | Arts-inclusive programs (programs for the general population).  Defined as being inclusive of any art form.  Can be passive or interactive engagement in the arts. | Programs/interventions without any arts exposure  For one-off programs/interventions (i.e., one single session): the duration of the arts exposure in less than two hours. |
| **Outcome** | Mental health and/or wellbeing outcome defined as broadly to include internalising symptoms, anxiety, depression, and socioemotional wellbeing.  Mental health and wellbeing outcome does not include behavioural problems, emotion regulation, aggression, self-esteem, emotion expression, relationship quality, agency, resilience, communication skills, inhibitory control, social skills and coping. However, these outcomes may be included if they form part of a validated scale to measure overall wellbeing or mental health (e.g., measuring ‘attention’ as a MH outcome in people with ADHD). | No mental health or wellbeing outcomes. |
| **Study characteristics** | Published date: 2014-present  In English  Peer-reviewed | Articles that cannot be retrieved.  Retracted articles  Non-peer-reviewed (book chapters, dissertations)  Review or opinion articles (including articles where methodology and results are not presented clearly) |

**Supplementary Table S3** *Inter-Rater Reliability for MMAT Quality Assessment*

| MMAT Criterion | Study Type | *n* | *κ* | % Agreement |
| --- | --- | --- | --- | --- |
| S1 | All | 36 | 1.00^a^ | 100.0 |
| S2 | All | 36 | 1.00 | 100.0 |
| 1.1 | Quantitative RCT | 6 | 1.00 | 100.0 |
| 1.2 | Quantitative RCT | 6 | 1.00 | 100.0 |
| 1.3 | Quantitative RCT | 6 | 1.00 | 100.0 |
| 1.4 | Quantitative RCT | 6 | 1.00 | 100.0 |
| 1.5 | Quantitative RCT | 6 | 1.00 | 100.0 |
| 2.1 | Quantitative non-randomised | 12 | 0.56 | 75.0 |
| 2.2 | Quantitative non-randomised | 12 | 1.00 | 100.0 |
| 2.3 | Quantitative non-randomised | 12 | 1.00 | 100.0 |
| 2.4 | Quantitative non-randomised | 12 | 0.84 | 91.7 |
| 2.5 | Quantitative non-randomised | 12 | 1.00 | 100.0 |
| 3.1 | Quantitative descriptive | 18 | 0.91 | 94.4 |
| 3.2 | Quantitative descriptive | 18 | 1.00 | 100.0 |
| 3.3 | Quantitative descriptive | 18 | 0.73 | 88.9 |
| 3.4 | Quantitative descriptive | 18 | 0.75 | 83.3 |
| 3.5 | Quantitative descriptive | 18 | 1.00 | 100.0 |
| 4.1 | Qualitative | 2 | 1.00 | 100.0 |
| 4.2 | Qualitative | 2 | 1.00 | 100.0 |
| 4.3 | Qualitative | 2 | 1.00 | 100.0 |
| 4.4 | Qualitative | 2 | 1.00 | 100.0 |
| 4.5 | Qualitative | 2 | 1.00 | 100.0 |
| 5.1 | Mixed methods | 5 | 1.00 | 100.0 |
| 5.2 | Mixed methods | 5 | 1.00 | 100.0 |
| 5.3 | Mixed methods | 5 | 1.00 | 100.0 |
| 5.4 | Mixed methods | 5 | 1.00 | 100.0 |
| 5.5 | Mixed methods | 5 | 1.00 | 100.0 |

*Note.* MMAT = Mixed Methods Appraisal Tool. For 14 criteria where both raters showed perfect agreement with no variance in ratings (S1, 1.1, 1.2, 1.3, 1.4, 1.5, 2.5, 4.2, 4.4, 4.5, 5.1, 5.3, 5.4, 5.5), Cohen's κ could not be calculated; 100% agreement is reported for these items.

**Supplementary Table S4** *Study Characteristics*

| **Study** | **Intervention Characteristic** | **Artistic Activity** | **Sample size (n)** | **Duration (weeks)** | **No. of sessions** | **Frequency (per week)** | **Session duration (min)** | **Setting** | **Trainer** | **Outcomes** | **Instrument** |
| --- | --- | --- | --- | --- | --- | --- | --- | --- | --- | --- | --- |
| 1. Archbell et al., 2019 (41) | Recreational | Dance, Multi Arts, Music | 166 | Not applicable  (Cross sectional study) | Not applicable  (Cross sectional study) | Not applicable  (Cross sectional study) | Not applicable  (Cross sectional study) | Not applicable  (Cross sectional study) | Not applicable  (Cross sectional study) | Emotional Regulation | Strengths & Difficulty Questionnaire |
|  |  |  |  |  |  |  |  |  |  | Externalising | Strengths & Difficulty Questionnaire |
|  |  |  |  |  |  |  |  |  |  | Other | Psychological engagement in performing arts |
|  |  |  |  |  |  |  |  |  |  | Social Skills | Strengths & Difficulty Questionnaire |
|  |  |  |  |  |  |  |  |  |  | Stress | Single item question |
| 2. Badura et al., 2015 (56) | Recreational | Visual Arts | 1953 | Not Reported | Not Reported | Not Reported | Not Reported | Out of school hours | Not Reported | Wellbeing | Life satisfaction scale |
| 3. Boal-Palheiros & Ilari, 2023 (37) | Recreational | Drama, Music | 56 | 30 | 30 | 1 | 60 | School | Teacher in relevant field | Social Skills | Social Skills Rating System- social skills composite |
|  |  |  |  |  |  |  |  |  |  | Internalising | Social Skills Rating System- internalising |
|  |  |  |  |  |  |  |  |  |  | Externalising | Social Skills Rating System- externalising |
|  |  |  |  |  |  |  |  |  |  | Externalising | Hyperactivity scale |
| 4. Bokoch & Hass-Cohen, 2021 (38) | Therapeutic | Visual Arts | 56 | 8 | 8 | 1 | 60 | School | Art Therapist in relevant field | Externalising | Symptoms and Functioning Severity Scale |
|  |  |  |  |  |  |  |  |  |  | Wellbeing | CORS |
|  |  |  |  |  |  |  |  |  |  | Internalising | Symptoms and Functioning Severity Scale |
| 5. Brouzos et al., 2016 (55) | Therapeutic | Multi arts | 48 | 6 | 6 | 1 | 90 | Community | Researcher | Wellbeing | Emotional wellbeing scale |
|  |  |  |  |  |  |  |  |  |  | Social Skills | Social anxiety scale |
| 6. Brovchak et al., 2024 (57) | Recreational | Multi Arts | 64 | 26 | 24 | 4 | Not reported | School | School Teacher | General Mental Health | Strengths & Difficulty Questionnaire |
|  |  |  |  |  |  |  |  |  |  | Social Skills | Strengths & Difficulty Questionnaire |
|  |  |  |  |  |  |  |  |  |  | Other | Non-verbal abstract reasoning |
| 7. Cheung et al., 2024 (36) | Recreational | Music | 32 | 24 | 24 | 1 | 60 | Community | Artist | Other | Interviews |
|  |  |  |  |  |  |  |  |  |  | Depression | Center for Epidemiologic Studies Depression Scale (CES-D) |
| 8. Conover, 2020 (42) | Therapeutic | Storytelling | 36 | 6 | Variable | 3 | Variable | Home | Parents | Externalising | Child behavioural checklist |
|  |  |  |  |  |  |  |  |  |  | Other | Resiliency |
| 9. Davies et al., 2023 (43) | Recreational | Music | 27 | 2 | 10 | 5 | 20 | School | School Teacher | Wellbeing | Focus group |
|  |  |  |  |  |  |  |  |  |  | Stress | Focus group |
| 10. Gerami, 2021 (45) | Therapeutic | Visual Arts | 10 | 10 | 10 | 1 | 120 | School | Art Therapist in relevant field | Stress | Perceived Stress Scale |
|  |  |  |  |  |  |  |  |  |  | Biomarker | Cortisol levels in saliva |
|  |  |  |  |  |  |  |  |  |  | General Mental Health | Symptom Checklist 90 Revised |
|  |  |  |  |  |  |  |  |  |  | Wellbeing | KINDL-R (QOL) |
| 11. Hinshaw et al., 2015 (52) | Recreational | Music | 50 | 26 | Not reported | Not Reported | Not reported | School | Researcher | Wellbeing | Stirling Wellbeing Scale |
|  |  |  |  |  |  |  |  |  |  | General Mental Health | Strengths & Difficulty Questionnaire |
|  |  |  |  |  |  |  |  |  |  | Emotional Regulation | Interviews |
| 12. Jo et al., 2018 (28) | Therapeutic | Visual Arts | 17 | 12 | 12 | 1 | 60 | Home | Healthcare Professional | Anxiety | Revised Children's Manifest Anxiety Scale |
|  |  |  |  |  |  |  |  |  |  | Depression | Draw-A-Story (DAS) Test |
| 13. Kang et al., 2021 (47) | Therapeutic | Visual Arts | 50 | 8 | 8 | 1 | 45 | School | Healthcare Professional | Biomarker | Attention quotient (ATQ) |
|  |  |  |  |  |  |  |  |  |  | Other | Self-esteem scale |
|  |  |  |  |  |  |  |  |  |  | Stress | Han's stress scale |
| 14. Kevers et al., 2022 (39) | Therapeutic | Multi Arts | 32 | 3 | 6 | 2 | 20 | online (video conferencing) | Researcher | Other | Youth Self-Report (YSR) PTSP scale |
|  |  |  |  |  |  |  |  |  |  | Internalising | Strengths & Difficulty Questionnaire |
|  |  |  |  |  |  |  |  |  |  | Externalising | Strengths & Difficulty Questionnaire |
| 15. Kim & Kim, 2018 (30) | Recreational | Music | 30 | 24 | 24 | 1 | 50 | School | Teacher in relevant field | Emotional Regulation | Emotional intelligence scale |
|  |  |  |  |  |  |  |  |  |  | Anxiety | Anxiety scale |
|  |  |  |  |  |  |  |  |  |  | Externalising | Aggression scale |
| 16. Kim & Lee, 2023 (31) | Therapeutic | Music | 36 | 5 | 10 | 2 | 45 | Community | Healthcare Professional | General Mental Health | Behavior Detection VibraImage System |
|  |  |  |  |  |  |  |  |  |  | Stress | Behavior Detection VibraImage System |
|  |  |  |  |  |  |  |  |  |  | Anxiety | Behavior Detection VibraImage System |
|  |  |  |  |  |  |  |  |  |  | Social Skills | Behavior Detection VibraImage System |
|  |  |  |  |  |  |  |  |  |  | Biomarker | Heart-rate variability |
| 17. Léger-Goodes et al., 2023 (29) | Therapeutic | Visual Arts | 81 | 10 | 10 | 1 | 45 | School | Researcher | Anxiety | BASC-3 Anxiety |
|  |  |  |  |  |  |  |  |  |  | Depression | BASC-3 Depression |
|  |  |  |  |  |  |  |  |  |  | Other | BASC-3 Inattention |
| 18. Malboeuf-Hurtubise et al., 2021 (25) | Therapeutic | Visual Arts | 30 | 3 | 6 | 2 | 20 | online (video conferencing) | Researcher | Depression | BASC-3 |
|  |  |  |  |  |  |  |  |  |  | Anxiety | BASC-3 Anxiety |
| 19. Merati et al., 2019 (44) | Recreational | Music | 8 | Not Reported | Not Reported | Not Reported | Not Reported | Not Reported | Not Reported | Stress | Focus group |
| 20. Milaré et al., 2021 (34) | Therapeutic | Storytelling | 111 | 8 | 16 | 2 | 30 | School | Teacher in relevant field | Stress | Childhood stress scale |
|  |  |  |  |  |  |  |  |  |  | Other | Five Digit Test |
|  |  |  |  |  |  |  |  |  |  | General Mental Health | Positive and Negative Affect Schedule for Children (NEGATIVE SUBSCALE) |
|  |  |  |  |  |  |  |  |  |  | Anxiety | Multidimensional Anxiety Scale for Children |
|  |  |  |  |  |  |  |  |  |  | Depression | Child Depression inventory |
| 21. Moula et al., 2020 (48) | Therapeutic | Multi Arts | 88 | 4 | 4 | 1 | 120 | School | Healthcare Professional | Biomarker | Physical health (Sleep duration) |
|  |  |  |  |  |  |  |  |  |  | General Mental Health | Strengths & Difficulty Questionnaire |
|  |  |  |  |  |  |  |  |  |  | Wellbeing | CORS |
|  |  |  |  |  |  |  |  |  |  | Wellbeing | EQ-5D-Y (QOL) |
| 22. Moula, 2021 (65) | Therapeutic | Dance, Drama, Multi Arts, Music, Visual Arts | 62 | 8 | 8 | 1 | 60 | School | Art Therapist in relevant field | Other | Interviews |
|  |  |  |  |  |  |  |  |  |  | Emotional Regulation  Other | Interviews  Interviews |
| 23. Moula et al., 2022 (50) | Therapeutic | Dance | 68 | 8 | 8 | 1 | 120 | School | Art Therapist in relevant field | Wellbeing | Health related quality of life Scale |
|  |  |  |  |  |  |  |  |  |  | Wellbeing | Life functioning & wellbeing Scale |
|  |  |  |  |  |  |  |  |  |  | Emotional Regulation | Semi-structured interviews |
| 24. Moula et al., 2023 (46) | Therapeutic | Visual Arts | 16 | 8 | 8 | 1 | 60 | School | Art Therapist in relevant field | Stress | EQ-5D-Y (QOL) |
|  |  |  |  |  |  |  |  |  |  | General Mental Health | Strengths & Difficulty Questionnaire |
|  |  |  |  |  |  |  |  |  |  | General Mental Health | CORS |
|  |  |  |  |  |  |  |  |  |  | Biomarker | Biomarker (Sleep) |
|  |  |  |  |  |  |  |  |  |  | Wellbeing | EQ-5D-Y (QOL) |
| 25. Moula et al., 2023 [b] (49) | Therapeutic | Visual Arts | 101 | 8 | 8 | 1 | A full day | School | Artist | Wellbeing | EQ-5D-Y (QOL) |
| 26. Pasiali & Clark, 2018 (40) | Therapeutic | Music | 20 | 10 | 8 | 1 | 50 | Out of school hours | Art Therapist in relevant field | Social Skills | Home & Community Social Behavioral Scale |
|  |  |  |  |  |  |  |  |  |  | Internalising | Social Skills Improvement System internalising |
|  |  |  |  |  |  |  |  |  |  | Externalising | Social Skills Improvement System externalising |
|  |  |  |  |  |  |  |  |  |  | Social Skills | Social Skills Improvement System social skills total |
| 27. Patil et al., 2023 (26) | Therapeutic | Visual Arts | 60 | 3 | 3 | Not Reported | Not reported | Clinic | Not applicable | Anxiety | Study group |
| 28. Pereira & Marques-Pinto, 2017 (51) | Therapeutic | Dance | 45 | 12 | 12 | 1 | 50 | School | Healthcare Professional | Wellbeing | Mental Health Continuum |
| 29. Pesso-Aviv et al., 2014 (33) | Recreational | Visual Arts | 13 | 10 | 10 | Not reported | Not reported | Camp | Researcher | Anxiety | Trait anxiety scale |
|  |  |  |  |  |  |  |  |  |  | Anxiety | Situation anxiety scale |
| 30. Rose et al., 2019 (32) | Recreational | Music | 19 | 40 | 40 | 1 | Not reported | School | Teacher in relevant field | Externalising | BAS-C Aggression |
|  |  |  |  |  |  |  |  |  |  | Internalising | BAS-C Internalising |
|  |  |  |  |  |  |  |  |  |  | Anxiety | BAS-C Anxiety |
| 31. Ruini et al., 2022 (27) | Recreational | Creative writing | 8 | Not Reported | Not Reported | 4 | Not reported | Out of school hours | Teacher in relevant field | Depression | Cognitive Triad Inventory for Children (CTI-C) |
|  |  |  |  |  |  |  |  |  |  | Wellbeing | Ryff’s Psychological Well-Being Scales (PWB) |
|  |  |  |  |  |  |  |  |  |  | Anxiety | Revised Children’s Manifest Anxiety Scale (RCMAS) |
| 32. Sitzer & Stockwell, 2015 (53) | Therapeutic | Visual Arts | 43 | 14 | 14 | 1 | 60 | School | Researcher | Wellbeing | Wellness Inventory, scales 26-182 |
| 33. Stephenson, 2023 (54) | Therapeutic | Drama | 30 | 40 | Not Reported | 1 | 90 | School | Researcher | Wellbeing | Positive feelings and wellbeing |
| 34. Tardif et al., 2024 (35) | Recreational | Music | 93 | Not Reported | Not Reported | Not Reported | Not reported | School | Teacher in relevant field | Anxiety | Music performance anxiety |

**Supplementary Table S5** *List of the measures used by the studies included in this review, organised by outcome*

| **Outcome** | **Measure** |
| --- | --- |
| Anxiety | Anxiety scale  BAS-C Anxiety  BASC-3 Anxiety  Behavior Detection VibraImage System  Multidimensional Anxiety Scale for Children  Music performance anxiety  Revised Children’s Manifest Anxiety Scale (RCMAS)  Situation anxiety scale  Study group  Trait anxiety scale |
| Depression | BASC-3 Depression  Center for Epidemiologic Studies Depression Scale (CES-D)  Child Depression inventory  Cognitive Triad Inventory for Children (CTI-C)  Draw-A-Story (DAS) Test |
| Externalising | Aggression scale  BAS-C Aggression  Child behavioural checklist  Hyperactivity scale  Social Skills Improvement System externalising  Social Skills Rating System- externalising  Strengths & Difficulty Questionnaire  Symptoms and Functioning Severity Scale |
| General Mental Health | Behavior Detection VibraImage System  CORS  Positive and Negative Affect Schedule for Children (NEGATIVE SUBSCALE)  Strengths & Difficulty Questionnaire  Symptom Checklist-90 Revised |
| Internalising | BAS-C Internalising  Social Skills Improvement System internalising  Social Skills Rating System- internalising  Strengths & Difficulty Questionnaire  Symptoms and Functioning Severity Scale |
| Stress | Behavior Detection VibraImage System  Childhood stress scale  EQ-5D-Y (QOL)  Focus group  Han's stress scale  Perceived Stress Scale  Single item question |
| Wellbeing | CORS  EQ-5D-Y (QOL)  Emotional wellbeing scale  Focus group  Health related quality of life Scale  KINDL-R (QOL)  Life functioning & wellbeing Scale  Life satisfaction scale  Mental Health Continuum  Positive feelings and wellbeing  Ryff’s Psychological Well-Being Scales (PWB)  Stirling Wellbeing Scale  Wellness Inventory, scales 26-182 |

**Supplementary Figure S6**.Critical appraisal of studies

a) Quantitative RCT b) Quantitative non RCT


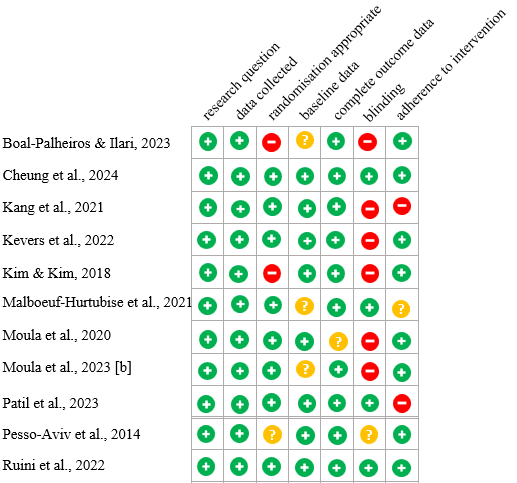

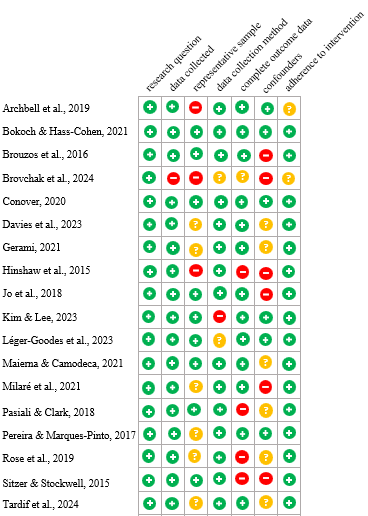


c) Qualitative d) Mixed Methods


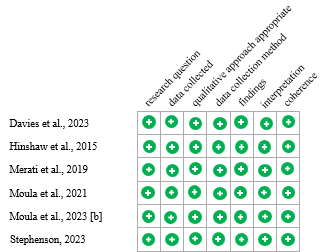

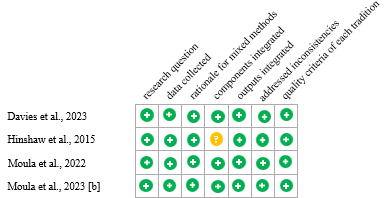


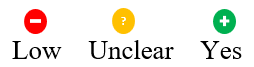


**Supplementary Figure S7.** Critical appraisal of quantitative studies


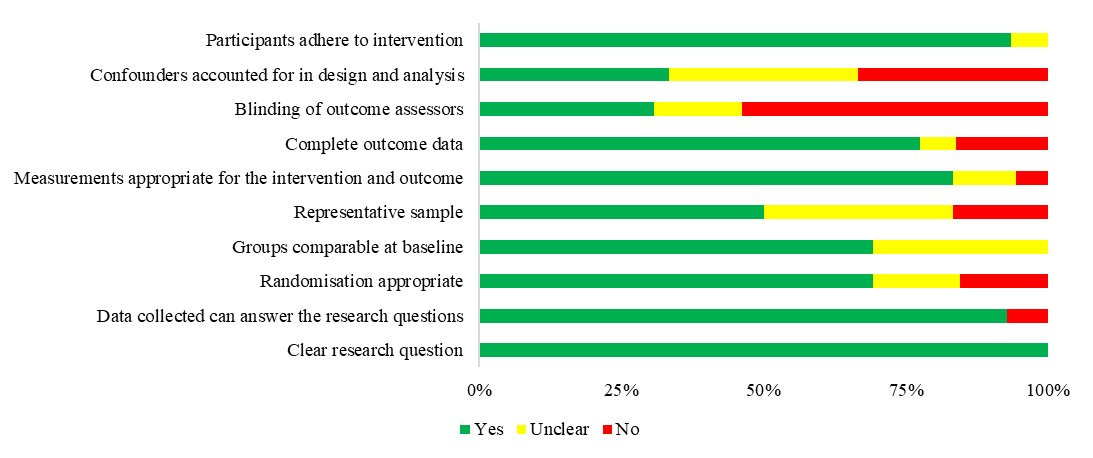


**Supplementary Table S8.** *GRADE evidence profile*

| **Outcome** | **Study design** | **Risk of bias** | **Inconsistency** | **Indirectness** | **Imprecision** | **publication bias** | **Certainty** |
| --- | --- | --- | --- | --- | --- | --- | --- |
| Internalising | RCT/non RCT | serious^a^ | serious^b^ | not serious | serious^c^ | strongly suspected |  |
| Anxiety | RCT/non RCT | not serious | serious^b^ | not serious | serious^c^ | not detected |  |
| Depression | RCT/non RCT | not serious | serious^b^ | not serious | serious^c^ | strongly suspected |  |
| Internalising (general) | RCT/non RCT | serious^a^ | serious^b^ | not serious | serious^c^ | strongly suspected |  |
| Externalising | RCT/non RCT | not serious | serious^b^ | not serious | serious^c^ | not detected |  |
| Processes | RCT/non RCT | serious^a^ | not serious | not serious | serious^c^ | not detected |  |
| Stress | RCT/non RCT | not serious | not serious | not serious | serious^c^ | not detected |  |
| Wellbeing | RCT/non RCT | serious^a^ | not serious | not serious | serious^c^ | not detected |  |
| General Mental Health | RCT/non RCT | serious^a^ | not serious | not serious | serious^c^ | strongly suspected |  |

*Note:* RCT: Randomised Control Trial; ^a^ inadequate blinding of participants; ^b^ inconsistent results across studies; ^c^ small sample sizes.

Certainty of evidence (GRADE):

High certainty The true effect is close to that of the estimate of the effect.

Moderate certainty The true effect is likely to be close to the estimate of the effect, but there is a possibility that it is substantially different.

Low certainty The true effect may be substantially different from the estimate of the effect.

Very low certainty The true effect is likely to be substantially different from the estimate of effect.

**Supplementary Table S9.** *GRADE-CERQual evidence profile*

| **Outcome** | **Study design** | **Methodological limitations** | **Relevance** | **Coherence** | **Data adequacy** | **Certainty** |
| --- | --- | --- | --- | --- | --- | --- |
| Processes | Qualitative | no concerns | no concerns | no concerns | serious concerns |  |
| Stress | Qualitative | no concerns | no concerns | no concerns | moderate concerns |  |
| Wellbeing | Qualitative | no concerns | no concerns | no concerns | serious concerns |  |

Certainty of evidence (GRADE):

High certainty The true effect is close to that of the estimate of the effect.

Moderate certainty The true effect is likely to be close to the estimate of the effect, but there is a possibility that it is substantially different.

Low certainty The true effect may be substantially different from the estimate of the effect.

Very low certainty The true effect is likely to be substantially different from the estimate of effect.

**Supplementary Table S10.** *Summary of mechanisms identified*

| **Domain** | **Mechanism** | **N Studies** | **Studies** | **Evidence Type** | **Associated Outcomes** |
| --- | --- | --- | --- | --- | --- |
| Biological | Sleep/Circadian regulation* | 3 | Moula et al., 2023 (45); Moula et al., 2022 (49); Moula et al., 2020 (47) | Theoretical | Wellbeing, General Mental Health, Stress |
| Biological | Reduced cortisol/HPA axis regulation | 1 | Gerami, 2021 (44) | Theoretical | Stress, Wellbeing, General Mental Health |
| Biological | Relaxation response | 1 | Moula et al., 2023 [b] (48) | Qualitative theme | Wellbeing |
| Biological | Embodied cognition | 1 | Stephenson, 2023 (53) | Qualitative theme | Wellbeing |
| Biological | Physical activity | 1 | Archbell et al., 2019 (40) | Theoretical | Stress, Externalising |
| Psychological | Self-efficacy/confidence | 3 | Moula et al., 2023 [b] (48); Stephenson, 2023 (53); Hinshaw et al., 2015 (51) | Qualitative theme | Wellbeing, General Mental Health |
| Psychological | Positive affect/enjoyment | 1 | Davies et al., 2023 (42) | Qualitative theme | Stress, Wellbeing |
| Psychological | Emotional expression | 1 | Stephenson, 2023 (53) | Qualitative theme | Wellbeing |
| Psychological | Stress reduction/relaxation | 1 | Davies et al., 2023 (42) | Qualitative theme | Stress, Wellbeing |
| Psychological | Self-esteem/self-worth | 1 | Stephenson, 2023 (53) | Qualitative theme | Wellbeing |
| Psychological | Agency/autonomy | 1 | Moula et al., 2023 [b] (48) | Qualitative theme | Wellbeing |
| Psychological | Coping skills/strategies | 1 | Cheung et al., 2024 (35) | Qualitative theme | Depression |
| Psychological | Meaning making | 1 | Moula et al., 2022 (49) | Qualitative theme | Wellbeing |
| Social | Sense of belonging/social inclusion | 1 | Davies et al., 2023 (42) | Qualitative theme | Stress, Wellbeing |
| Social | Collaboration/cooperation | 1 | Boal-Palheiros & Ilari, 2023 (36) | Theoretical | Internalising, Externalising |

*Note:* *Sleep quality/regulation added to framework; not in original Fancourt and colleagues article (20).

**Supplementary Table S11.** *Summary of Context-Mechanism-Outcomes identified*

| **Study** | **Context** | **Context** | **Context** | **Context** | **Context** | **Mechanisms** | **Outcomes** |
| --- | --- | --- | --- | --- | --- | --- | --- |
|  | **Art Form** | **Type** | **Duration** | **Setting** | **Facilitator** |  |  |
| Davies et al., 2023 (42) | Music | Recreational | 2 | School | School Teacher | Psychological: Positive affect/enjoyment (Qual theme) | Wellbeing (Beneficial) |
|  |  |  |  |  |  | Psychological: Stress reduction/relaxation (Qual theme) |  |
|  |  |  |  |  |  | Social: Sense of belonging/social inclusion (Qual theme) |  |
| Stephenson 2023 (53) | Drama | Therapeutic | 40 | School | Researcher | Psychological: Emotional expression (Qual theme) | Wellbeing (Beneficial) |
|  |  |  |  |  |  | Psychological: Self-efficacy/confidence (Qual theme) |  |
|  |  |  |  |  |  | Psychological: Self-esteem/self-worth (Qual theme) |  |
|  |  |  |  |  |  | Biological: Embodied cognition/somatic awareness (Qual theme) |  |
| Cheung et al., 2024 (35) | Music | Recreational | 24 | Community | Artist | Psychological: Coping skills/strategies (Qual theme) | Depression (Beneficial) |
| Moula et al., 2023 [b] (48) | Visual Arts | Therapeutic | 8 | School | Artist | Psychological: Self-efficacy/confidence (Qual theme) | Wellbeing (Beneficial) |
|  |  |  |  |  |  | Psychological: Agency/autonomy (Qual theme) |  |
|  |  |  |  |  |  | Biological: Relaxation response (Qual theme) |  |
| Hinshaw et al., 2015 (51) | Music | Recreational | 26 | School | Researcher | Psychological: Self-efficacy/confidence (Qual theme) | Wellbeing (Harmful) General Mental Health (Beneficial) |
| Moula et al., 2022 (49) | Dance | Therapeutic | 8 | School | Art Therapist in relevant field | Psychological: Meaning making (Qual theme) | Wellbeing (Beneficial) |
|  |  |  |  |  |  | Biological: Sleep/Circadian regulation* (Theoretical) |  |
| Gerami 2021 (44) | Visual Arts | Therapeutic | 10 | School | Art Therapist in relevant field | Biological: Reduced cortisol/HPA axis regulation (Theoretical) | Stress (Beneficial) Wellbeing (Beneficial) |
| Moula et al., 2023 (45) | Visual Arts | Therapeutic | 8 | School | Art Therapist in relevant field | Biological: Sleep/Circadian regulation* (Theoretical) | Wellbeing (Beneficial) General Mental Health (Beneficial) Stress (Beneficial) |
| Moula et al., 2020 (49) | Multi Arts | Therapeutic | 4 | School | Healthcare Professional | Biological: Sleep/Circadian regulation* (Theoretical) | Wellbeing (Beneficial) General Mental Health (Beneficial) |
| Archbell et al., 2019 (40) | Dance | Recreational | Not applicable* | Not applicable* | Not applicable* | Biological: Physical activity/movement (Theoretical) | Externalising (Beneficial) |
| Boal-Palheiros & Ilari, 2023 (36) | Music | Recreational | 30 | School | Teacher in relevant field | Social: Collaboration/cooperation (Theoretical) | Internalising (Beneficial) Externalising (Beneficial) |

*Note:* *Cross sectional study
